# Non-small cell lung cancer (NSCLC): characteristics, risk factors, molecular profile patterns, and treatment — a retrospective cohort study from Palestine

**DOI:** 10.1186/s43046-025-00298-8

**Published:** 2025-07-14

**Authors:** Abdallah Damin Abukhalil, Khaldieh Mansour, Wardah Alhaj, Inas Salah, Yousef Sahoury, Ni’meh Al-Shami, Mohammad Qawasmeh

**Affiliations:** Birzeit University, Birzeit, Palestinian Territory

**Keywords:** Non-small cell lung cancer, Overall survival, PDL expression, Immunotherapy, Chemotherapy, ECOG performance status

## Abstract

**Introduction and background:**

Non-small cell lung cancer (NSCLC) is the third most common type of cancer in Palestine and has the highest mortality rate. Treatment approaches for NSCLC depend on many factors including stage, histology, molecular profile, and patient performance status.

**Objectives:**

This study explored the patient characteristics, molecular profiles, metastatic sites, prognosis, and treatment modalities.

**Methods:**

This observational retrospective cohort study was conducted at multiple Palestinian hospitals. This study included patients diagnosed with metastatic NSCLC between 2016 and 2022. Patients with small-cell lung cancer (SCLC), newly diagnosed lung cancer, or incomplete information were excluded from the study. Patient data were obtained from the date of lung cancer diagnosis until death or loss to follow-up. Data were analyzed using IBM SPSS, and overall survival was calculated using the Kaplan–Meier estimate.

**Results:**

The study included 102 patients, 80.4% were male, 40.2% were current smokers, 42.2% were ex-smokers, and 17.6% were nonsmokers. (86.35%) of the patients were diagnosed with adenocarcinoma, and (77.5%) were diagnosed with stage IV NSCLC. Tumor recurrence was observed in 47.1% of patients after surgery. A total of 56.9% had PDL-1 expression ≥ 10%, and 45.1% had EGFR mutations. Fourteen (13.7%) received mono-chemotherapy with an estimated OS of (1219.200) days, 34 (33.3%) received mono-immunotherapy with an estimated OS of (720.152) days, and 54 (52.9%) received a combination of chemotherapy and immunotherapy with an OS of 2006.777 days. PFS (> 1 year) was higher in patients receiving combination therapy (58.3%). Myelosuppression, renal damage, and liver damage are some of the major side effects experienced by patients receiving either type of treatment.

**Conclusion:**

The findings of this study provide vital information on tumor molecular mutation patterns and PDL expression for the adoption of appropriate measures in prevention and treatment strategies for NSCLC in Palestine. The majority of patients diagnosed with NSCLC were males with a history of smoking and were diagnosed at an advanced stage, which requires increased education, wariness of lung cancer, and smoking cessation programs at the national level.

## Introduction

Lung cancer is the third most common type of cancer in Palestine, with the highest mortality rate, and a high percentage of lung cancer patients have a history of smoking or are currently smoking, according to the 2019 latest statistics [1]. On the basis of histology, lung cancer is classified into small cell lung cancer (SCLC) and non-small cell lung cancer (NSCLC), including adenocarcinoma, squamous carcinoma, and large cell carcinoma [2]. Treatment approaches for lung cancer depend on many factors including stage, histology, genetic mutations, and patient performance status [3]. Treatment options for lung cancer include monotherapy or a combination of different modalities such as surgery, radiation, chemotherapy, immunotherapy, and molecular target therapy.

Treatments are also based on the expression of programmed cell death protein–ligand 1 on the surface of malignant cells (PD-L1) and the type of genetic mutation, which differs from one patient to another. PD-L1 expression has been used as a biomarker for immune checkpoint inhibitor therapy responses. PD-L1, also known as programmed cell death ligand 1, is a transmembrane protein expressed on the surface of tumor cells, specifically in lung cancer, breast cancer, melanoma, and some normal human immune cells. Immunotherapy is preferred when PD-L1 expression is more than 10% and is optimized when it is more than 50%, whereas less than 1% PD-L1 limits the use of immunotherapy [4]. The identification of genetic mutations in NSCLC, including mutations in the epidermal growth factor receptor (EGFR) with mutation types including exons (18, 19, 20, 21), L858R, T790M, anaplastic lymphoma kinase (ALK), ROS-1 (v-ros UR2 sarcoma virus oncogene homolog 1), v-Raf murine sarcoma viral oncogene homolog B1 (BRAF), and Kirsten rat sarcoma viral oncogene homolog (KRAS), has led to the development of tyrosine kinase inhibitors to target these mutations [5]. The emergence of immunotherapy has provided desirable results in the treatment and management of metastatic NSCLC compared with traditional chemotherapy [6].

Traditional chemotherapeutic agents, such as alkaloids, taxanes, anthracyclines, and alkylating agents, interfere with the cellular synthesis of DNA, RNA, cell growth, and cell division. Platinum has been the standard modality in the management of NSCLC [7]. The National Comprehensive Cancer Network (NCCN) recommends that histologic subtype workup be the determining factor in selecting the treatment modalities in patients with metastatic NSCLC, because targeted therapy has been shown to decrease tumor burden, decrease symptoms, and improve the quality of life for patients with specific genomic alterations. Furthermore, if molecular testing results are unavailable and patients require an urgent start of therapy, clinicians may hold immunotherapy for one cycle and start platinum-based chemotherapy regimens [8]. Access to healthcare and the availability of resources are significant barriers to healthcare services in Palestine, and governmental oncology units lack specialized pathology laboratories and diagnostic testing, and chemotherapy is offered as the main treatment [9]. The current management of treatment protocols for metastatic NSCLC in Palestine includes protocols that use a combination of chemotherapy and immunotherapy as a second-line treatment. For metastatic NSCLC, chemotherapeutic agents include carboplatin, cisplatin, paclitaxel, pemetrexed, docetaxel, and gemcitabine, using one or a combination of these agents once a week for a certain amount of time, depending on the patient’s cancer stage and overall response, with a complete blood workup performed before each cycle. Immunotherapeutic agents, including nivolumab, pembrolizumab, atezolizumab, and pertuzumab, were administered at least once every 21 days for a maximum of 2 years. To the best of our knowledge, no studies have been conducted in Palestine to explore the molecular patterns and characteristics of NSCLC.

This explanatory cohort study aimed to describe the characteristics, biomarkers, metastatic sites, and prognosis of patients with NSCLC in Palestine. Furthermore, the study aims to compare the effectiveness and safety of mono-chemotherapy, mono-immunotherapy, and the combination of chemoimmunotherapy in terms of overall survival (OS), progression-free survival (PFS), and adverse effects.

## Methods

### Study design

This observational, retrospective cohort study reviewed the electronic medical charts of patients diagnosed with non-small cell lung cancer (NSCLC) at two tertiary hospitals in Palestine, Augusta Victoria Hospital and Beit Jala Hospital, between 2016 and 2022. Patients with small-cell lung cancer (SCLC), newly diagnosed lung cancer, or incomplete information were excluded from the study. Patient data were obtained from the date of lung cancer diagnosis until death or loss of follow-up. Data of all patients who met the inclusion criteria were extracted, yielding a total of 102 patients.

A data collection form was designed to retrieve patient information from oncology departments in Palestinian hospitals. The form was divided into six sections: patient demographics (age, gender, and smoking history), patient performance (ECOG, efficacy response, disease-free survival, overall survival, and tumor recurrence), cancer histology, genetic profile (type of NSCLC, tumor node metastasis type, site of metastasis, PDL-1 expression, and other mutations), previous and current cancer treatments, side effects from treatment, and neutrophil–lymphocyte ratio. Data were collected by three 5 th-year PharmD students with the aid of an oncology specialist and were reviewed by two oncology clinical pharmacists.

### Statistical analysis

Collected data were added to an Excel spreadsheet version 16.71 and separated, coded, and analyzed using the Statistical Package for the Social Sciences (IBM SPSS version 29). Analyzing the data and interpreting the categorical data into frequencies and percentages, mean, median, and interquartile ranges were used for continuous data. The chi-square test and Fisher’s exact test were performed to determine the association between the study variables and the type of current treatment expressed by the *P*-value, showing a significant association when the *P*-value was less than 0.05. Overall survival and the type of treatment were calculated using the Kaplan–Meier estimate. The sample size was not calculated because of the limited number of documented cases of diagnosed metastasized non-small cell lung cancer in Palestine.

### Ethical considerations

This study was approved by the Ethics Committee of Birzeit University (reference number: BZU-PNH-2305). Personal information was not collected, and participants’ identities remained unknown. All the collected information was used for research purposes. The research was performed in accordance with relevant guidelines and regulations. The Birzeit Ethical Committee did not require consent to participate in the study because this was a retrospective study conducted by collecting data from medical charts.

## Results

### Patient characteristics and demographics

Table 1 shows an overview of the participants’ characteristics and pharmacological treatment modalities. This study included 102 patients who met the inclusion criteria: 82 were male (80.4%), and 20 were female (19.6%). Of the 102 patients, 41 (40.2%) were current smokers, 43 (42.2%) were ex-smokers, and 18 (17.6%) were nonsmokers.

**Table 1:** Patient demographics, cancer molecular structure, and prior therapy use

	*N*		*n* (%)	Combination	Chemotherapy	Immunotherapy
Gender	102	Male	82 (80.4%)	43 (52.4%)	9 (11.0%)	30 (36.6%)
		Female	20 (19.6%)	11 (55.0%)	5 (25.0%)	4 (20.0%)
Age	102	< 50 years old	21 (20.6%)	10 (47.6%)	2 (9.5%)	9 (42.9%)
		51–60 years old	26 (25.5%)	16 (61.5%)	4 (15.4%)	6 (23.1%)
		61–70 years old	33 (32.4%)	16 (48.5%)	4 (12.1%)	13 (39.4%)
		> 70 years old	22 (21.6%)	12 (54.5%)	4 (18.2%)	6 (27.3%)
Performance status (ECOG)	102	0	13 (12.7%)	5 (38.5%)	2 (15.4%)	6 (46.2%)
		1	30 (29.4%)	23 (76.7%)	5 (16.7%)	2 (6.7%)
		2	29 (28.4%)	21 (72.4%)	6 (20.7%)	2 (6.9%)
		≥ 3	30 (29.4%)	5 (16.7%)	1 (3.3%)	24 (80.0%)
Tumor recurrence after surgery	102	Yes	48 (47.1%)	26 (54.2%)	9 (18.8%)	13 (27.1%)
		No	31 (30.4%)	22 (71.0%)	0 (0%)	9 (29.0%)
		No surgery	23 (22.5%)	6 (26.1%)	5 (21.7%)	12 (52.2%)
Histology type of NSCLC	102	Adenocarcinoma	88 (86.3%)	49 (55.7%)	12 (13.6%)	27 (30.7%)
		Squamous cell	11 (10.8%)	3 (27.3%)	1 (9.1%)	7 (63.6%)
		Large cell	3 (2.9%)	2 (66.7%)	1 (33.3%)	0 (0%)
Tumor node metastasis	102	IIIB	15 (14.7%)	9 (60.0%)	3 (20.0%)	3 (20.0%)
		IIIC	8 (7.8%)	6 (75.0%)	2 (25.0%)	0 (0%)
		IVA	41 (40.2%)	23 (56.1%)	1 (2.4%)	17 (41.5%)
		IVB	38 (37.3%)	16 (42.1%)	8 (21.1%)	14 (36.8%)
PDL-1 expression	94	< 1%	12 (11.8%)	5 (41.7%)	3 (25.0%)	4 (33.3%)
		1–10%	24 (23.5%)	15 (62.5%)	4 (16.7%)	5 (20.8%)
		≥ 10%	58 (56.9%)	30 (51.7%)	5 (8.6%)	23 (39.7%)
Genetic mutation	-	EGFR mutation	46 (45.1%)	27 (58.7%)	5 (10.9%)	14 (30.4%)
		ALK	3 (2.9%)	1 (33.3%)	1 (33.3%)	1 (33.3%)
		ROS-1	14 (13.7%)	8 (57.1%)	3 (21.4%)	3 (21.4%)
		BRAF	3 (2.9%)	2 (66.7%)	0 (0%)	1 (33.3%)
		KRAS	19 (18.6%)	11 (57.9%)	2 (10.5%)	6 (31.6%)
		Other	3 (2.9%)	1 (33.3%)	2 (66.7%)	0 (0%)
EGFR mutation type	-	Exon 18	0 (0%)	0 (0%)	0 (0%)	0 (0%)
		Exon 19	7 (6.9%)	4 (57.1%)	0 (0%)	3 (42.9%)
		Exon 20 T790M	11 (10.8%)	8 (72.7%)	0 (0%)	3 (27.3%)
		Exon 21 L858R	25 (24.5%)	14 (56.0%)	4 (16.0%)	7 (28.0%)
Prior chemotherapy use	102	Yes	46 (45.1%)	24 (52.2%)	4 (8.7%)	18 (39.1%)
		No	56 (54.9%)	30 (53.6%)	10 (17.9%)	16 (28.6%)

^*^Others: Napsin A, CK7, CDX2, TTF-1, CK20

### Tumor histology, staging, and mutation

A total of 86.35% of the patients were diagnosed with adenocarcinoma. Moreover, 77.5% of patients were diagnosed with stage IV NSCLC. PDL-1 expression ≥ 10% was observed in 56.9% of the patients. EGFR mutations were the most common type of mutations spread among patients (45.1%), and exon 21 (L858R) was the most common type of EGFR mutation (24.5%).

### Pharmacotherapy treatment modalities

Immunotherapy was the most common type of treatment among patients with an ECOG score ≥ 3 (80.0%), compared to 46.2%, 6.7%, and 6.9% of patients with ECOG scores of 0, 1, and 2, respectively. Tumor recurrence after surgery was observed in 48 patients (47.1%), of which 26 received combination therapy, and 71.0% of patients who did not experience tumor recurrence after surgery received combination therapy.

The combination of chemotherapy and immunotherapy was the most commonly used treatment for varying PDL-1 expression percentages, genetic mutations, and EGFR mutations.

### Metastatic sites

**Fig. 1:**
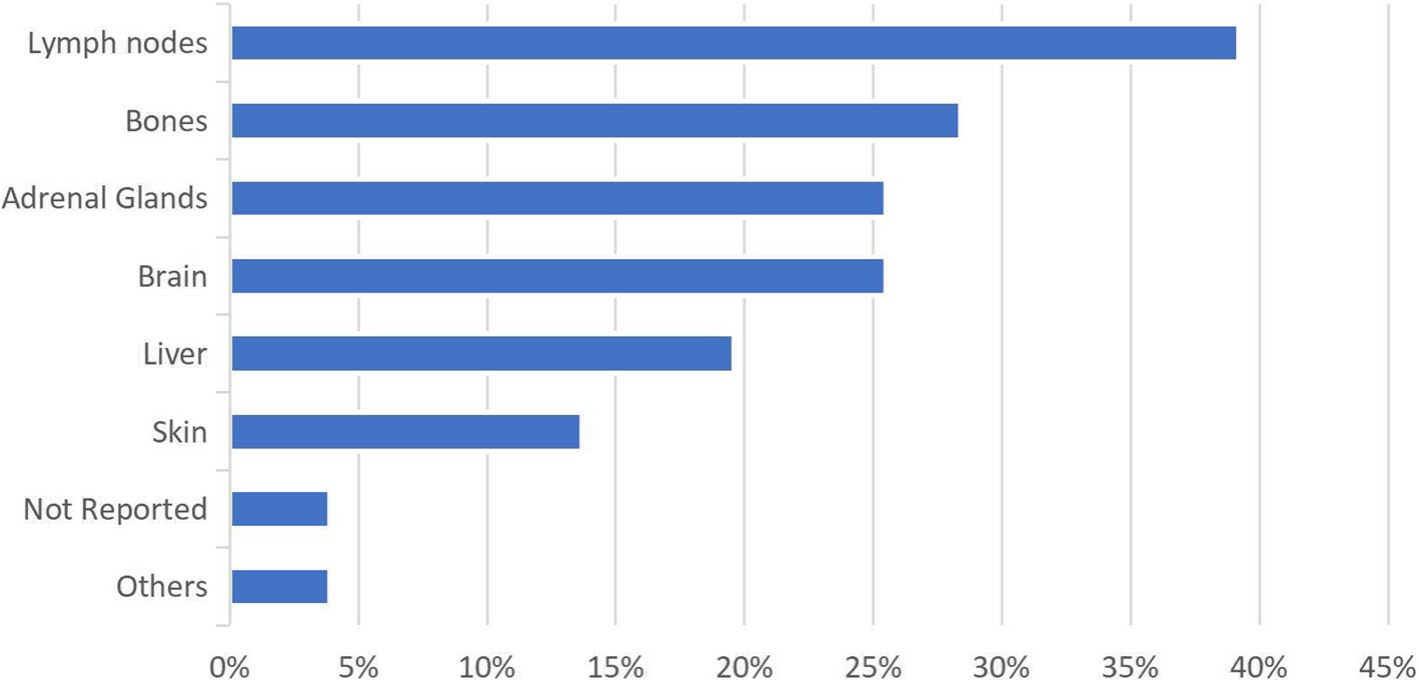
Sites of metastasis

Figure 1 shows the sites of metastasis: lymph nodes (39.2%), bones (28.4%), brain (25.5%), and adrenal glands (25.5%) were the most common sites of metastasis.

The distribution of all the current therapies is shown in Table 2, with paclitaxel/docetaxel, pemetrexed, and pembrolizumab being the most frequently utilized chemotherapy-immunotherapy combinations.

**Table 2:** Pharmacological treatment modalities

**Treatment type**	%	Agents	*n* (%)
Chemotherapy	14 (13.7%)	Folate antagonist	1 (1%)
		Platinum + taxane	5 (4.9%)
		Platinum + antimetabolite	1 (1%)
		Platinum + folate antagonist	3 (2.9%)
		Taxane + antimetabolite	1 (1%)
		Antimetabolite + folate antagonist	3 (2.9%)
Immunotherapy	34 (33.3%)	Nivolumab	9 (8.8%)
		Pembrolizumab	25 (24.5%)
Chemotherapy and immunotherapy (combination)	54 (52.9%)	Taxane + nivolumab	1 (1.0%)
		Platinum + antimetabolite + nivolumab	2 (2.0%)
		Platinum + antimetabolite + pembrolizumab	7 (6.9%)
		Platinum + taxane + pembrolizumab	4 (3.9%)
		Platinum + taxane + nivolumab	1 (1.0%)
		Platinum + folate antagonist + nivolumab	5 (4.9%)
		Platinum + folate antagonist + pembrolizumab	5 (4.9%)
		Platinum + folate antagonist + pertuzumab	1 (1.0%)
		Antimetabolite + pembrolizumab	5 (4.9%)
		Antimetabolite + nivolumab (Opdivo)	1 (1.0%)
		Antimetabolite + folate antagonist + nivolumab	4 (3.9%)
		Taxane + folate antagonist + pembrolizumab	8 (7.8%)
		Taxane + antimetabolite + pembrolizumab	7 (6.9%)
		Taxane + antimetabolite + nivolumab	3 (2.9%)

### Patient prognosis

Table 3 shows the association between the type of treatment and the patient’s efficacy response, which was determined using the chi-squared test. Most patients who received combination therapy (35.3%) showed a partial response and stable response (33.3%), and a smaller proportion of patients (30.4%) showed a progressive response, with a *P*-value of 0.0028, showing an association between these two variables.

**Table 3:** Treatment responses

Variable	Category	*n* (%)	Combination	Chemotherapy	Immunotherapy	*p*-value
Response at 3 months of treatment (101)	Complete response	0 (0%)	0 (0%)	0 (0%)	0 (0%)	0.019
	Partial response	36 (35.3%)	19 (52.8%)	8 (22.2%)	9 (25.0%)	
	Stable response	34 (33.3%)	21 (61.8%)	0 (0%)	13 (38.2%)	
	Progressive disease	31 (30.4%)	13 (41.9%)	6 (19.4%)	12 (38.7%)	
PFS (102)	≤ 1 year	66 (64.7%)	33 (50.0%)	13 (19.7%)	20 (30.3%)	0.053
	> 1 year	36 (35.3%)	21 (58.3%)	1 (2.8%)	14 (38.9%)	

### Adverse effects

**Fig. 2:**
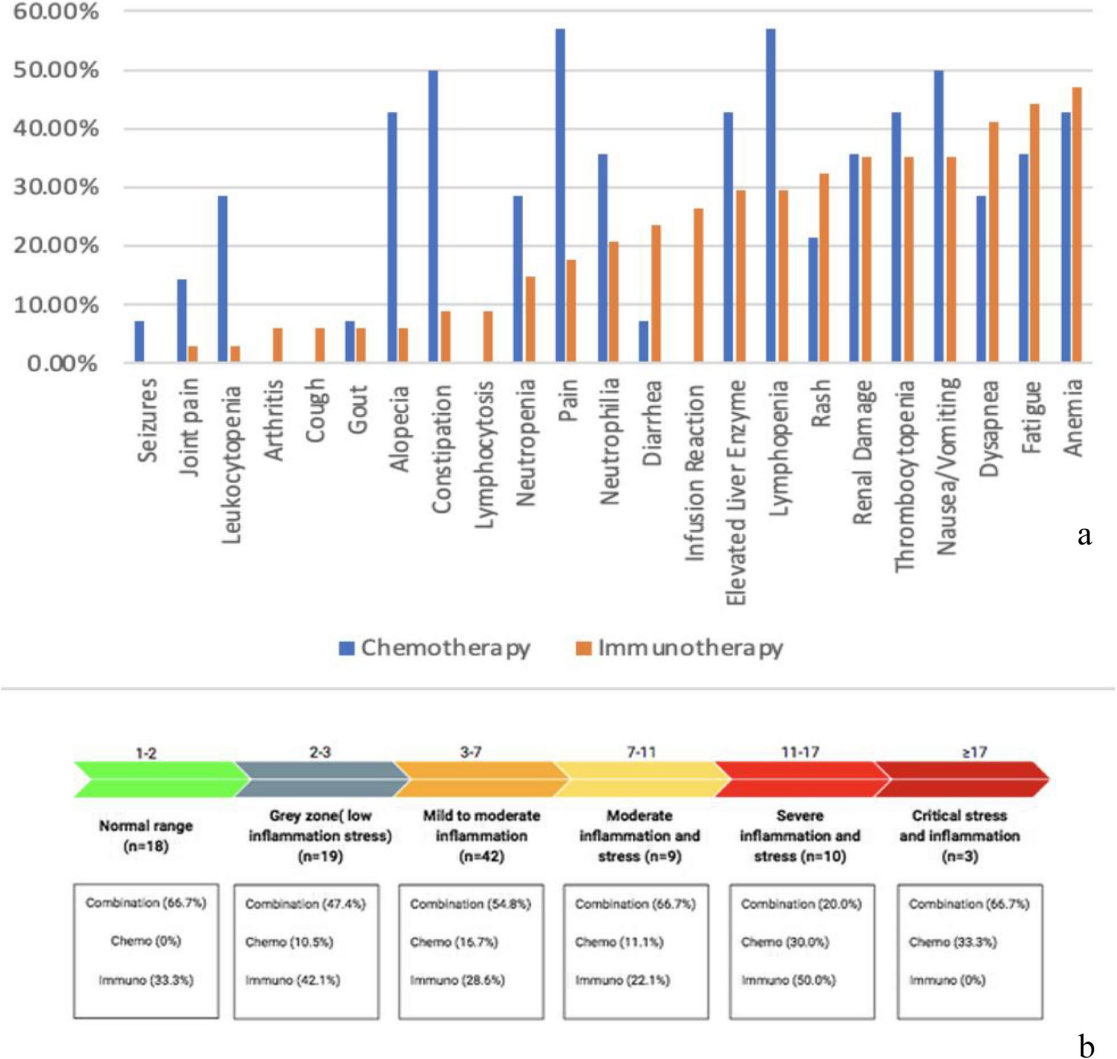
**a** Experienced treatment side effects. **b** NLR range of the participant

Figure 2a shows the percentage of common side effects experienced by the patients who were administered either chemotherapy or immunotherapy. The data analysis revealed a significant association between treatment type and the occurrence of leukocytosis, infusion reaction, constipation, alopecia, and pain with *P*-values of 0.021, 0.043, 0.003, 0.005, and 0.013, respectively, and Fig. 2b shows the NLR range and the type of treatment used by the patients. The statistical analysis gave a *P*-value of 0.195 showing that there was no association between the variables.

### Survival analysis

**Fig. 3:**
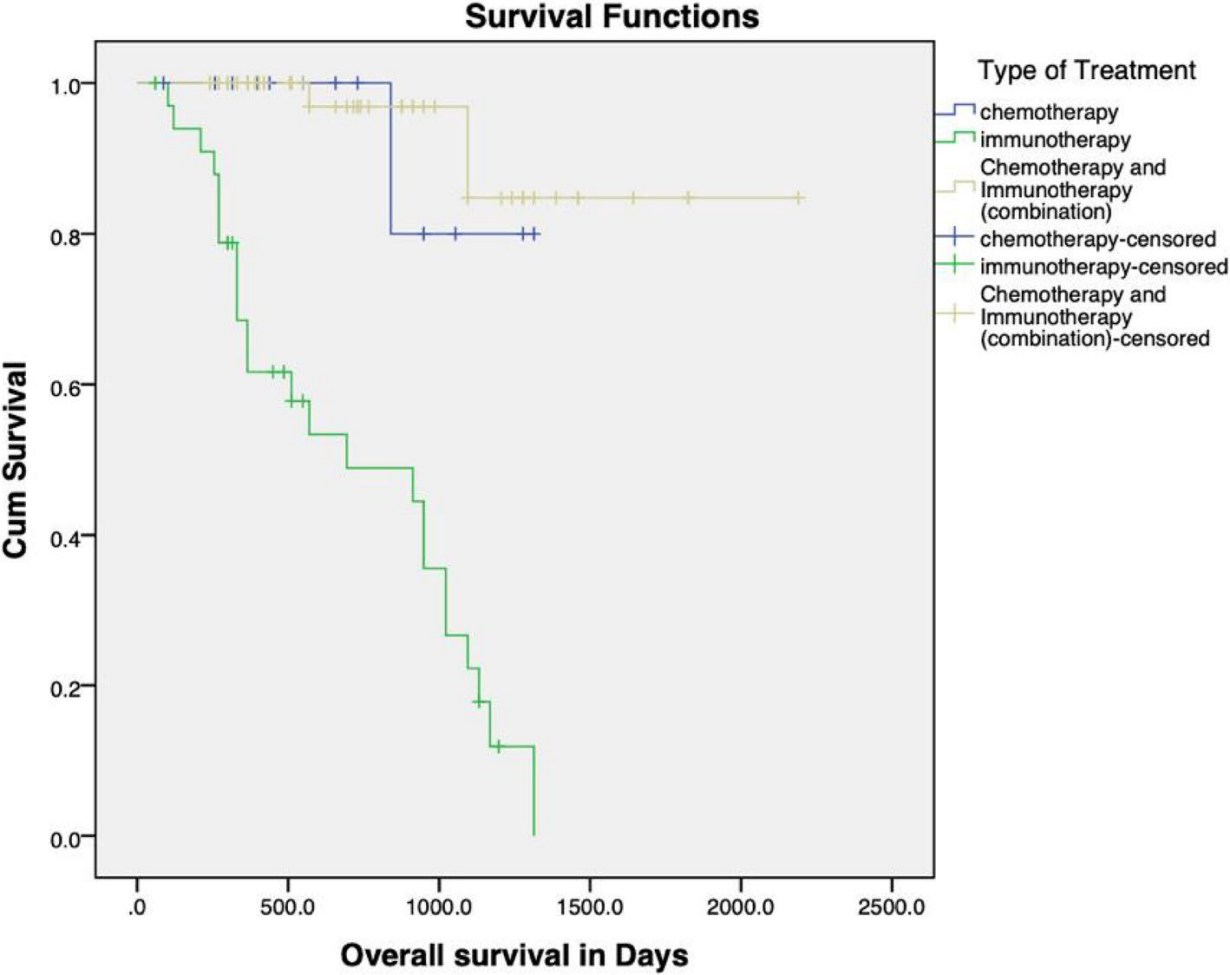
Overall survival and type of current treatment

Figure 3 displays the patients’ OS based on Kaplan–Meier analysis, with a significant difference in the estimate of the overall survival among the different types of therapy (*P*-value < 0.001).

The estimated mean survival times of 2006.777 (95% *CI* = 1813–2200.5), 1219.200 (95% *CI* = 1053–1385.4), and 720.152 (95% *CI* = 569.3–871.1) days were observed in patients who received combination therapy, chemotherapy, and immunotherapy, respectively.

## Discussion

In this study, we explored the characteristics, biomarkers, treatments, and progression of patients diagnosed with NSCLC and explored various elements affecting therapy determination for advanced stages and metastatic non-small cell lung cancer in Palestine.

In this study, it is evident that the number of males (80.4%) diagnosed with NSCLC was much higher than that of females, which is very similar to the findings of a study in Palestine and in other regional countries in Africa and the Middle East KINDLE study, where more than 80% of patients diagnosed with NSCLC were males and approximately 50% had adenocarcinoma [10, 11]. Many factors increase the risk of lung cancer among Palestinian males. Smoking has been reported to be more prevalent in males in Palestine owing to socioeconomic and cultural factors. It has been reported that the rate of smoking exceeds 36% among adults in Palestine [11]. A multicenter study on the prevalence of tobacco use in Palestine revealed that tobacco smoking was higher among males than females by a factor of 2 [12]. This finding was supported by the results of several previous studies. The higher incidence of lung cancer in males is due to their excessive smoking history, and smokers have a higher risk of developing lung cancer in their lifetime [13].

Furthermore, a systematic review and meta-analysis revealed that smoking cessation at or around the time of diagnosis was significantly associated with improved overall survival in patients with NSCLC, SCLC, or lung cancer [14]. Adenocarcinoma has been reported as the most prevalent histological subtype of lung cancer, which was evident in this study, as the most common histological subtype in the study was adenocarcinoma (86.3% of the participants had lung adenocarcinoma). Studies of NSCLC conducted in Jordan, Lebanon, and the USA have reported that adenocarcinoma is the most common type of NSCLC [15–17].

Programmed cell death protein 1 (PD-1) and programmed cell death ligands are among the most important biomarkers in treatment decision-making. PD-1/PDL-1 inhibitors, such as nivolumab and pembrolizumab, achieve their therapeutic effect by preventing the antigen binding of PD-1 and PDL-1, allowing T cells to attack and destroy tumor cells [18]. In this study, 56.9% of the patients had PDL-1 expression, which is very similar to a recent study conducted in Lebanon, which revealed a PDL expression of 61.7% [17]. In this study, patients with PDL expression who received immunotherapy as monotherapy or in combination therapy had a better overall response than patients with low or no PDL-1 expression. In the present study, we observed that a higher efficacy response when immunotherapy was used in the presence of high PDL-1 expression was very evident in this study [19]. This finding is supported by a systematic review showing that PD-1/PD-L1 inhibitors are effective and promising immunotherapeutic agents for the treatment of many types of cancer [20]. Meanwhile, 11.8% of patients in the study had (PDL-1 expression < 1%), whereas 33.3% of them received immunotherapy regardless of the absence of PDL-1 expression due to a lack of other options, the patient’s performance status, inability to tolerate chemotherapy side effects, or the main aim of treatment being remission and not cure [21, 22]. In addition to PD-1/PDL-1 expression, EGFR mutations are known to become a part of the treatment modalities for patients with NSCLC. The EGFR mutation was found to be the most abundant genetic mutation type (45.1%), with exon 21 L858R being the most common EGFR subtype (24.5%). In a systematic review and meta-analysis of EGFR mutation frequency in Middle East and African non-small cell lung cancer patients, EGFR mutation exon 19 was the most prevalent type [23]. A clinical report performed in Jordan on patients with adenocarcinoma NSCLC showed that the prevalence of EGFR mutations is higher among Asian patients than among Jordanians and lower among African Americans [19]. The prevalence of EGFR mutations in this study was much higher than that in regional countries, such as Lebanon (12%), Morocco (21%), Egypt (27%), and the Gulf area (more than 30% in the Gulf area [24]. Combination therapy has been utilized in more than 50% of patients with EGFR mutations and is recommended to improve overall outcomes [25].

Tumor nodes affect treatment selection. In advanced stages, the treatment goal is to improve patients’ quality of life and overall survival. In this study, 22.5% of the participants had stage III NSCLC, and 77.5% had stage IV NSCLC. Most patients with advanced-stage disease were treated with combination therapy. In total, 56.1% of patients with stage IVA and 42.1% of patients with stage IVB received combination therapy, and the use of immunotherapy with chemotherapy improved the overall survival for advanced and metastatic stages of NSCLC [26].

### Pharmacotherapy treatment modalities

Patient performance status, or Eastern Cooperative Oncology Group (ECOG) performance status, is an important variable used in selecting treatment modalities to determine treatment and for optimum results, considering the patient’s tolerability and physical status. In this study, 46.2% of patients with an ECOG score of 0 received monotherapy, patients with ECOG 1 (76.7%) and ECOG 2 (72.4%) received combination therapy, and the majority of patients with poor performance status (ECOG ≥ 3) (80.0%) received monotherapy (Table 1). These findings are supported by the Clinical Oncology Guidelines for managing NSCLC in patients with an ECOG performance status ≥ 3. Chemotherapy can result in lower response rates and higher toxicity. These findings were supported by the ASCO clinical guidelines for the management of NSCLC in patients with an ECOG score ≥ 3 to use immunotherapy alone. The use of chemotherapy in patients with ECOG ≥ 3 can result in lower response rates and high toxicity [27], as found in our study; only one patient with ECOG ≥ 3 used chemotherapy alone, whereas other studies suggest that immunotherapy improves the quality of life and benefits the patient’s survival rate [28]. ECOG performance status can decline or improve in patients during the treatment period, explaining why patients with poor performance status receive chemotherapy or combination therapy.

Patients with early-stage resectable NSCLC can be treated or cured surgically by removing the affected tissue from their lungs [29]. In terms of late diagnosis at advanced or metastatic stages, surgical intervention for complete tumor resection is not recommended. However, surgery can reduce the tumor burden at the sites of metastasis [30]. Of the patients, 77.5% underwent surgery with the aim of tumor removal, and 22.5% had no surgery because of extended metastasis of the disease. Although surgery is a reliable option for early-stage cancer, some patients develop tumor recurrence mainly due to micrometastatic cancer cells [31]. In this study, 47.1% of the patients who underwent surgery experienced tumor recurrence. After recurrence, the tumor was most likely more aggressive and spread to other regions, requiring the addition of immunotherapy and targeted therapy to traditional chemotherapy, according to the National Cancer Institute (NCI) guidelines [32].

Many treatment options for lung cancer, including chemotherapy, are used if the patient can tolerate side effects, such as immunotherapy, which is used in the presence of PDL-1 expression. According to the American Cancer Society, targeted therapy and immunotherapy are the first-line agents for patients diagnosed with stage IVB NSCLC. According to the ASCO of Clinical Oncology, stages IIIB and C are treated using platinum-based doublet chemotherapy, an EGFR inhibitor, and a PDL-1 inhibitor (if EGFR mutations and PDL-1 expression are present). For stages IVA and B, first-line treatment includes chemotherapy with a PDL-1 inhibitor [33]. The combination of chemotherapy and immunotherapy has a synergistic effect, emphasizing its antitumor activity [34]. In Palestine, there is limited access to newer medications, and not all immunotherapy and targeted therapy, such as nivolumab, pertuzumab, and pembrolizumab, are available to patients [11]. Patients with EGFR mutations who are not treated with EGFR-TKI inhibitors, such as erlotinib or gefitinib, are considered first-line agents in these patients owing to their increased survival rates; however, these medications are not available [35]. In this study, only 4.9% of the patients received a combination of platinum-based chemotherapy with taxane, 24.5% of the patients received pembrolizumab, and 7.8% received a combination of taxane and folate antagonist chemotherapy with pembrolizumab immunotherapy. The limitations of cancer treatment options, diagnosis, and screening negatively impact patient outcomes. As shown in this study, the majority of patients were diagnosed in the later stages of cancer, making treatment options very challenging and resulting in a low survival rate. In Palestine, there is a lack of effective programs focusing on screening and controlling risk factors for cancer prevention. Furthermore, the limitations of pathological tools for treatment and diagnosis have caused many cancer patients to be referred to or seek treatment outside the territory, limiting access to care in conflict zones where movement is restricted and requires a permit that is often denied by the occupation authority [36].

### Site of metastasis

The most common sites of metastasis in NSCLC include the bone, brain, and abdominal regions (liver, adrenal glands, kidneys, and gastrointestinal tract) and lymph node involvement [37]. Data analysis revealed that 28.4% of the patients had bone metastasis, 25.5% had brain metastasis, 25.5% had adrenal gland metastasis, and 39.2% had lymph node metastasis. Studies have suggested that bone metastasis reduces the efficacy of immunotherapy when used alone, thus recommending combination therapy [37]. In this study, 48.3% of the patients with bone metastasis received a combination of immunotherapy and chemotherapy, and 41.4% received monotherapy. Studies have suggested that bone metastasis reduces the efficacy of immunotherapy when used alone [38], suggesting the use of combination therapy, as hypothesized by a Lebanese study [39].

### Patient prognosis

The efficacy of the selected treatment was determined by monitoring the tumor response 3 months after initiating treatment [40]. Complete response is very rare in metastatic lung cancer cases, considering the possibility of multiple site metastasis and late diagnosis [41], and as seen in the results, 0% of patients had no complete response. Most patients showed a partial and stable response, especially those receiving combination therapy (52.8% and 61.8%, respectively), indicating the extent of the therapy in tumor regression and stabilization of the disease from further growth, better outcomes, and fewer side effects [42]. Among patients with disease progression, 41.9% received combination therapy, and 38.7% received immunotherapy. The data analysis suggested that combination therapy has higher efficacy in tumor regression and disease stabilization than other therapies such as chemotherapy and immunotherapy monotherapy.

Progression-free survival is one of the main indicators of treatment efficacy and patient response to treatment. Thus, comparing the PFS associated with multiple treatments can yield promising outcomes, leading to the preferred treatment [43]. The data analysis of this study showed that patients receiving combination therapy as the main treatment (58.3%) had a higher PFS (> 1) than those receiving chemotherapy as monotherapy (19.7%), who had a lower PFS (≤ 1). The findings of improved PFS in patients receiving combination therapy are supported by clinical trials in Spain [44].

### Overall survival

Overall survival (OS) is one of the most important outcomes of treatment. Figure 3 shows that patients who received a combination of chemotherapy and immunotherapy had an estimated mean survival of 2006.777 days, while patients who received mono-chemotherapy and mono-immunotherapy survived with a mean of 1219.200 and 720.152 days, respectively. This analysis revealed that the combination regimen had the most prolonged overall survival, followed by mono-chemotherapy and mono-immunotherapy. These results are confirmed by many previous studies, which state that using chemotherapy and immunotherapy combinations provides better outcomes and responses and improves survival in patients with NSCLC compared to monotherapy [45], regardless of PDL1 expression [42, 43, 46]. In a five-year follow-up of immunotherapy versus chemotherapy in metastatic NSCLC, pembrolizumab showed a median OS of 26.3 months vs 13.4 months in the chemotherapy group. Furthermore, the immunotherapy group experienced fewer adverse events [47].

### Side effects

Nausea, vomiting, alopecia, fatigue, myelosuppression, and nephrotoxicity are the common side effects of chemotherapy [48]. Abdelazeem et al. [45] The side effects of immunotherapy include infusion reactions, rash, arthritis, muscle or joint aches, diarrhea, and fatigue [49]. The results of the present study show that nausea and vomiting were experienced by 50.0% of patients who received mono-chemotherapy compared to 35.30% of patients who received mono-immunotherapy. Nausea and vomiting are the side effects most likely caused by chemotherapy, and immunotherapy can also cause nausea and vomiting in patients, specifically nivolumab, as stated in other studies [50]. Alopecia was reported in 49.20% of patients receiving chemotherapy and 5.90% of patients receiving immunotherapy, indicating that alopecia is mainly associated with chemotherapy. Other studies suggested that alopecia may occur in patients receiving immunotherapy [51]. Infusion reactions are immunotherapy-mediated side effects that have been reported in several other studies [52]. Leana [49] In this study, 29.60% of patients receiving immunotherapy experienced an infusion reaction compared with 0% of those who received chemotherapy. Myelosuppression and hepatotoxicity were the other side effects that patients complained of regarding chemotherapy in this study, and the most common side effects and toxicities of chemotherapy were nausea, vomiting, alopecia, fatigue, myelosuppression, and nephrotoxicity [48]. However, regarding other side effects, immunotherapy patients complained of rash, which was the most common immune-mediated side effect, supported by studies that suggested that the side effects of immunotherapy include infusion reactions, rash, arthritis, muscle or joint aches, diarrhea, and fatigue [49]. When both agents are combined, there is a risk and severity of side effects, and toxicity increases [53], such as hematotoxicity, hepatotoxicity, and GI abnormalities [46].

### Limitations

The main limitation of this study is its small sample size of 102 patients, which limits the generalization of the study to treatment groups; however, it sets the groundwork for future large-scale studies with larger sample sizes. Furthermore, sampling coverage bias may have occurred since the study only involved two hospitals, excluding patients with limited access to healthcare and those who transferred to be treated outside the country, further limiting the representation of patients with NSCLC. Hospital documentation lacks long-term follow-up and survival data, which could have affected the results. Another limitation is inadequate genetic testing or a lack of genetic data, as not all patients were tested for PDL-1 expression and other genetic mutations affecting treatment selection and outcomes.

## Conclusion

This study highlights for the first time the essential observations of patients diagnosed with NSCLC in Palestine. Most patients are diagnosed at metastatic stages, calling for action measures at the national level to increase awareness of early diagnosis and preventive measures. Furthermore, regardless of tumor staging, histology, and mutations, a combination of chemotherapy and immunotherapy resulted in improved disease-free and overall survival, and patients exhibited a better response to treatment, suggesting that combination therapy should be applied as a first-line therapy for the treatment of advanced and metastatic lung cancer, as evidenced by this study. Despite the increased risk and severity of side effects, combination therapy is still favored over other treatment options, and the benefits outweigh the risks.

## Data Availability

The datasets used and/or analyzed during the current study are available from the corresponding author upon reasonable request.
